# Ubiquitous conservative interaction patterns between post-spliced introns and their mRNAs revealed by genome-wide interspecies comparison

**DOI:** 10.3389/fgene.2023.1151703

**Published:** 2023-04-12

**Authors:** Suling Bo, Qiuying Sun, Zhongxian Li, Gerile Aodun, Yucheng Ji, Lihua Wei, Chao Wang, Zhanyuan Lu, Qiang Zhang, Xiaoqing Zhao

**Affiliations:** ^1^ College of Computer Information, Inner Mongolia Medical University, Hohhot, China; ^2^ Department of Oncology, Inner Mongolia Cancer Hospital and the Affiliated People’s Hospital of Inner Mongolia Medical University, Hohhot, China; ^3^ Inner Mongolia Academy of Agricultural and Animal Husbandry Sciences, Hohhot, China; ^4^ School of Life Science, Inner Mongolia University, Hohhot, China; ^5^ Key Laboratory of Black Soil Protection and Utilization (Hohhot), Ministry of Agriculture and Rural Affairs, Hohhot, China; ^6^ Inner Mongolia Key Laboratory of Degradation Farmland Ecological Restoration and Pollution Control, Hohhot, China; ^7^ College of Science, Inner Mongolia Agriculture University, Hohhot, China

**Keywords:** local matched alignment, optimal matched region, interaction patterns, ubiquitous conservative, gene expression

## Abstract

Introns, as important vectors of biological functions, can influence many stages of mRNA metabolism. However, in recent research, post-spliced introns are rarely considered. In this study, the optimal matched regions between introns and their mRNAs in nine model organism genomes were investigated with improved Smith–Waterman local alignment software. Our results showed that the distributions of mRNA optimal matched frequencies were highly consistent or universal. There are optimal matched frequency peaks in the UTR regions, which are obvious, especially in the 3′-UTR. The matched frequencies are relatively low in the CDS regions of the mRNA. The distributions of the optimal matched frequencies around the functional sites are also remarkably changed. The centers of the GC content distributions for different sequences are different. The matched rate distributions are highly consistent and are located mainly between 60% and 80%. The most probable value of the optimal matched segments is about 20 bp for lower eukaryotes and 30 bp for higher eukaryotes. These results show that there are abundant functional units in the introns, and these functional units are correlated structurally with all kinds of sequences of mRNA. The interaction between the post-spliced introns and their corresponding mRNAs may play a key role in gene expression.

## 1 Introduction

Since introns, a kind of non-coding DNA, were discovered, there have been many investigations of their functions and evolutionary origin (Roy, 2003). A research study recognized that the main function of introns is alternative splicing, facilitating the expression of multiple proteins from a single gene (Daehyun and Phil, 2005). Recently, it has become increasingly clear that introns are very important vectors of biological functions (Mattick and Gagen, 2001; Nott et al., 2003; Bianchi et al., 2009; Charital et al., 2009), and the sequence structures of introns and behavior of introns when removed by spliceosomes can influence many stages of mRNA metabolism (Orphanides and Reinberg, 2002; Hir et al., 2003). Many experiments have shown that introns can boost gene expression (Buchman and Berg, 1988; McKenzie and Brennan, 1996). Intron-containing transgenes in mice are transcribed 10–100 times more efficiently than their intron-less counterparts (Brinster et al., 1988), and the transcription of intron-less mRNA *in vivo* directs this mRNA toward translational silencing, while mRNA translational efficiency is dramatically increased by the addition of just one generic intron to the pre-mRNA (Callis et al., 1987). Although some genes contain no introns or their expressions do not require introns, introns can still improve the gene expression of genetically modified organisms (Duncker et al., 1997; Ko et al., 1998). It has also been discovered that the two small introns of the *Drosophila affinidisjuncta* (Adh) gene are required for normal transcriptions (Braddock et al., 1994). Intron mutation can cause many diseases. Besides the mutation at each end (GU and AG), the mutation in the middle of the intron sequences can also cause diseases by activating recessive splice sites (Stover and Verrelli, 2010; Nordin et al., 2012).

An increasing body of evidence shows that there are many introns in the cytoplasm and that they directly regulate gene translational efficiency. Intron sequences are retained in a number of dendritically targeted mRNAs in the cytoplasm (Buckley et al., 2011). Certain spliced mRNAs can be efficiently exported and translated, whereas the same mRNA transcribed from cDNA fails to exit the nucleus and express protein (Ryu and Mertz, 1989; Rafiq et al., 1997; Matsumoto et al., 1998). Removal of an intron from a pre-mRNA, without significantly altering the steady-state cytoplasmic mRNA level, can also affect translational efficiency (Luo and Reed, 1999). Similarly, when a mature mRNA is injected directly into oocyte nuclei, the translation efficiency is repressed and overcome by either adding a spliced intron or injecting FRGY2 protein antibodies into the cytoplasm (Hir et al., 2003). In addition, it is interesting to note that introns can suppress RNA silencing in *Arabidopsis* (Christie et al., 2011).

Many experiments have proved that introns function significantly in all processes of regulating the dynamic structure of mRNAs, their transport and nuclear export, and translation and regulation (Guigó and Ullrich, 2020; Gozashti et al., 2022). However, how introns take part in these biological processes is still unclear. In the past, it was believed that most pre-mRNAs were spliced to liner molecules with only exons. However, circular RNAs (circRNAs) were discovered, showing that the exon–circRNA model is formed by lariat-driven cyclization and intron-paired cyclization (Hansen et al., 2011; Jeck et al., 2013; Sebastian et al., 2013) and circular intronic RNAs can be formed by introns as well (Julia et al., 2012).

Based on these observations, it is believed that introns can directly affect gene expression after splicing by their interactions with the corresponding mRNAs. These kinds of interactions can maintain and regulate mRNA structures. The loss/gain of an intron does affect gene expression after splicing and plays a very important role in the evolution of the eukaryotic genome and the presence of new eukaryotic species (Duret, 2001; Halligan and Keightley, 2006). The interaction between post-spliced introns and their CDS was studied in our early works (Zhao et al., 2013; Zhang et al., 2016; Bo et al., 2019), but the 5′-UTR and 3′-UTR of mRNA are very important to gene expression. It is therefore very meaningful to study the interaction between introns and their corresponding mRNAs and to uncover how introns influence stages of gene expression after splicing by their interactions. Here, we report on the interaction characters between post-spliced introns and their mRNAs in whole genomes.

## 2 Materials and methods

### 2.1 Gene sequences

Genes from nine model organism genomes were selected as our dataset. They are *Caenorhabditis elegans*, *Drosophila melanogaster*, *Apis mellifera*, *Anopheles gambiae*, *Arabidopsis thaliana*, *Oryza sativa*, *Danio rerio*, *Mus musculus*, and *Homo sapiens*, and their gene sequences were downloaded from the Beijing Multi Subnet of Gene Bank (ftp://ftp.cbi.pku.edu.cn/pub/database/genomes). In this dataset, the genes that contain more than one mRNA were excluded first. Next, the genes that contain ncRNAs and/or repetitive elements were excluded. Last, introns with lengths shorter than 40 bp were also excluded. The results of the dataset are shown in Table 1.

**TABLE 1:** Genes of nine eukaryotes.

	Chromosome	Number of genes	Number of introns
*Caenorhabditis elegans*	1	956	4,052
*Drosophila melanogaster*	1	1,322	3,846
*Arabidopsis thaliana*	1	3,311	16,822
*Apis mellifera*	1	439	2,894
*Anopheles gambiae*	1	2,238	7,919
*Oryza sativa*	1	594	2,905
*Danio rerio*	1	1,005	8,563
*Mus musculus*	1	1,126	10,117
*Homo sapiens*	1	1,194	9,265

### 2.2 Matched alignment

**FIGURE 1:**
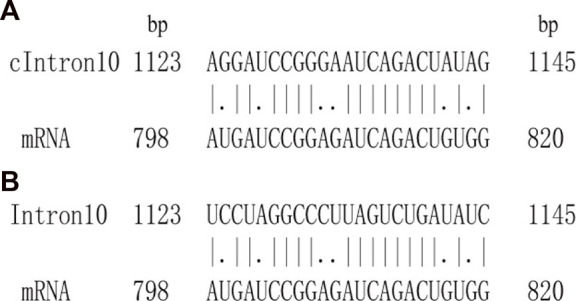
Sketch matched map between intron and corresponding CDS. **(A)** Smith–Waterman local alignment. cIntron10 means the complementary segment of intron 10. **(B)** Authentic matched alignment.

The interaction between introns and their mRNAs was represented by optimal matched segments. The interaction probability was determined by the quality of the optimal matched segments. The mRNAs were renamed as tested sequences, while their corresponding introns were aligned sequences. To obtain the matched alignment segments, introns were transformed into their complementary sequences, and similar alignments were performed using improved Smith–Waterman local alignment software (http://mobyle.pasteur.fr/cgi-bin/). In the alignment process, the EDNAFULL matrix was used for calculating the optimal matched segments with the following parameters: 50.0 for the gap penalty and 5.0 for extend penalty. In this way, the most credible optimal matched segment of the tested sequence and its aligned sequence were obtained. The local alignment sketch map is shown in Figure 1.

For the tested sequence, the matched score function *f* is defined by Eq. 1.

$$f=\left\{\begin{array}{cc} 1 & N_{s}\leq j\leq N_{e}\\ 0 & j<N_{s}\;or\;j>N_{e} \end{array}\right.,$$ (1)

where *j* means the *j*th base site of the tested sequence (*j* = 1,2, … , *L*), *L* means the length of the tested sequence, and *N*_*s*_ and *N*_*e*_ mean the start base site and the end base site of the optimal matched segment in the tested sequence, respectively. The effective value 1 is assigned to each base site within the optimal matched segment, while the ineffective value 0 is assigned to the base sites outside the optimal matched segment. The matched score values are assigned to each base site in the tested sequences.

For the tested sequences, matched frequency *F* is defined by Eq. 2.

$$F=1/m\sum_{i=1}^{m}f_{ij},$$ (2)

where *m* means the number of the tested sequences, *i* means the *i*th tested sequence (*i* = 1,2, … , *m*), *j* means the *j*th base site of the *i*th tested sequence (*j* = 1, 2, … , *L*_*i*_), *L*_*i*_ means the length of the *i*th tested sequence, and *f*_*ij*_ means the matched score function of the *j*th base site of the *i*th tested sequence. *F* is a relative matched value at the *j*th base site in *m* tested sequences. It reflects the interacting probability or the interaction intensity between the tested and aligned sequences in the *j*th base site.

The average matched frequency <*F*> for each base site is also defined by Eq. 3.

$$\langle F\rangle =1/m\sum_{i=1}^{m}l_{i}/L_{i}.$$ (3)

Here, *i* means the *i*th tested sequence (*i* = 1, 2, … , *m*), *l*_*i*_ is the length of the optimal matched segment for the *i*th tested sequence, and *L*_*i*_ is the length of the *i*th tested sequence. The <*F*> indicates the average matched frequency of the *m* tested sequences.

The relative matched frequency *RF* of the *j*th base site in the tested sequence is defined by Eq. 4.

$$RF=F/\langle F\rangle .$$ (4)

Here, *RF* reflects the relative bias of each base site in the tested sequences. If *RF* > 1, it indicates that the interaction in the *j*th base site is positive in the tested sequence, and the regions with *RF* > 1 were optimal matched regions. *RF* = 1 represents an average matched frequency of the base sites for the tested sequences.

To test the significance of our results, we constructed corresponding component constraint random sequences for comparison with real sequences. Component constraint random sequences mean the length and contents of A, C, G, and U are the same as the analyzed sequence, but the order of each base is random. We call them CC-random sequences. The sample of the corresponding component constraint random sequences is 10 times as many as the analyzed sequences, and then the corresponding *RF* or *F* distributions are obtained in the same way. When the *RF* values in the optimal matched regions of the mRNA all are higher than the CC-random sequences and average matched frequency <*F*>, we call these cases positive tests.

### 2.3 Sequence normalization

Due to the different lengths of the tested sequences, they are normalized to 100 to obtain the relative site distributions of *RF* or *F* by the following method.

We hypothesized that *n*_*ij*_ is the *j*th relative site of the *i*th normalized tested sequence; the *n*_*ij*_ is obtained by the following formulation:

$$n_{ij}=\left\{\begin{array}{cc} \left[100N_{ij}/L_{i}\right] & 100N_{ij}/L_{i}is\;integer\\ \left[100N_{ij}/L_{i}\right] & 100N_{ij}/L_{i}is\;non-integer \end{array}\right..$$ (5)

Here, *N*_*ij*_ means the *j*th base site of the *i*th tested sequence, and *L*_*i*_ is the length of *i*th tested sequence (*i* = 1, 2, …, *m*; *j* = 1, 2, …, *L*_*i*_). The square brackets are Gaussian integer functions which are meant to take the integer part of a real number. Then, the *m* tested sequences with different lengths are normalized to 100. In addition, n_*is*_, n_*ie*_, n_*ie*_^-^ n_*is*_+1, n_*ij*_ and 100 are used to replace N_*is*_, N_*ie*_, l_*i*_, N_*ij*_ and L_*i*_ in the formulation (1), (2), (3) and (4) respectively, the normalized relative matching frequency function *RF* or matching frequency function *F* distribution can be obtained.

### 2.4 Information entropy analysis

Information entropy conception was used to analyze the characters of sequence composition. Second-order informational redundancy *D*_*2*_ is a suitable parameter to describe the sequence characters; its definition is shown in Eq. 6.

For an analyzed sequence, the second-order informational redundancy *D*_2_ is defined as

$$D_{2}=\sum p_{ij}\log_{2}\left(p_{ij}/p_{i}p_{j}\right)\approx 1/2\ln2\sum {\left(p_{ij}-p_{i}p_{j}\right)}^{2}/p_{i}p_{j},$$ (6)

where *p*_*i*_ or *p*_*j*_ is the probability of the base *i* or *j* (*i, j* = A, C, G, U), and *p*_*ij*_ is the joint probability of the base pair *ij*. *D*_*2*_ reflects the adjacent base correlation of sequences (Luo and Hong, 1991; Li, 1990). In other words, a bigger *D*_*2*_ value means that the sequence is more conservative. For a finite sequence of length *N*, the fluctuation bound (f.b.) of *D*_*2*_ is *D*_*2*_(f.b.) = 15.65/*N* (Luo and Hong, 1991; Luo, 2004). When *D*_*2*_ ≥ 15.65/*N*, the neighboring bases do not occur independently, and the correlation exists at a 99% confidence level. Generally, *D*_*2*_ ≥ 0. For infinite random sequences, *D*_*2*_ = 0.

## 3 Results and discussion

### 3.1 Distributions of optimal matched regions in mRNA

**FIGURE 2:**
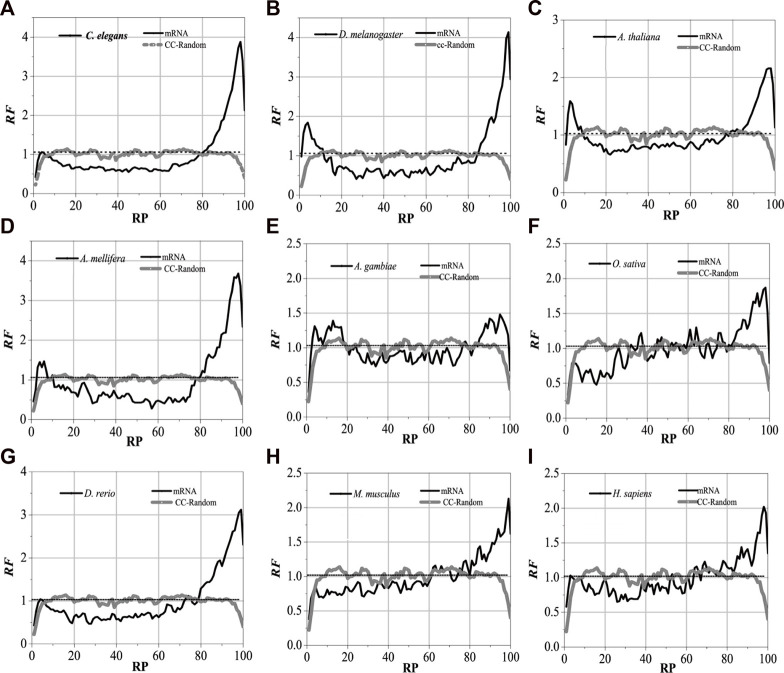
*RF* distributions of mRNA. The X-axis is the relative position of mRNA and the Y-axis represents the *RF* values. CC-Random means the local alignment were done between the component constraint random mRNA and their own component constraint random introns. *RF* = 1 represents the average value of relative match frequencies theoretically.

For the nine model organisms, mRNAs are regarded as the tested sequences, and their corresponding introns are regarded as the aligned sequences. The matched alignments between the mRNAs and their corresponding introns were performed, and the *RF* distributions with base relative sites of mRNA sequences were obtained (Supplementary Appendix SA1). Meanwhile, the local alignments were also performed between the component constraint random mRNAs and their own component constraint random introns, and they were marked as CC-random (Supplementary Appendix SA2). The results are shown in Figure 2.

The relative matched frequency distributions of the mRNA sequences of the nine model organisms were very similar to each other, which meant that the interaction patterns between the introns and their mRNAs are universal. When compared with the CC-random group, their characteristics are as follows: there are high relative matched frequencies in the UTR regions but a relatively low matched degree in the central protein-coding sequence. The relative matched frequency distributions of 3′-UTR are significantly higher than those of 5′-UTR (Supplementary Appendix SA3). It is speculated that the function of post-spliced introns is related to NMD. Compared with those in higher organisms, the matched frequency distributions in the mRNAs of lower eukaryotes are slightly different, and the distribution difference between the coding sequences and the UTR regions in lower organisms is more obvious, which reflects that the interaction modes between introns and their mRNAs in higher organisms are more complex.

Despite the species being very similar to each other for the matched frequency distributions in the mRNAs of the nine model organisms, the distributions of peak regions and peak values are slightly different (Figure 2). For *C. elegans*, the peak regions of the matched frequency distribution in the mRNAs are mainly located in 3%–8% of the 5′-end and between 80% and 98% of the 3′-end of the mRNA; the peaks are approximately 1.1 and 3.9, respectively. The peak regions for *D. melanogaster* are primarily found in 2%–10% of the 3′-end and 85%–99% of the 5′-end of the mRNA; the peak values are about 1.8 and 4.2, respectively. The peak regions for *A. thaliana* are mainly located in 2%–10% of the 5′-end and 85%–98% of the 3′-end of the mRNA, and the peak values are about 1.6 and 2.3, respectively. The peak regions for *A. mellifera* are mainly located in 2%–10% of the 5′-end and 80%–98% of the 3′-end of the mRNA, and the peak values are about 1.5 and 3.7, respectively. The peak regions of the distribution of *A. gambiae* are mainly located in 2%–20% of the 5′-end and 82%–98% of the 3′-end of the mRNA, respectively, and both peak values are about 1.4. The peak regions for *O. sativa* are mainly located in 80%–98% of the 3′-end of the mRNA, and the peak value is about 1.8. The peak regions for *D. rerio* are mainly in the 5%–8% of the 5′-end and 78%–99% of the 3′-end of the mRNA, and the peak values are about 1.1 and 3.1, respectively. The peak regions for *M. musculus* are mainly located in 80%–99% of the 3′-end of the mRNA, and both peak values are about 2.2. The peak regions for *H. sapiens* are distributed in 5%–10% of the 5′-end and 62%–99% of the 3′-end of the mRNA, and the peaks are about 1.1 and 2.0, respectively. It is suggested that introns have a strong preference for interactions with the corresponding mRNAs.

### 3.2 Matched characteristics of optimal matched segments of introns

**FIGURE 3:**
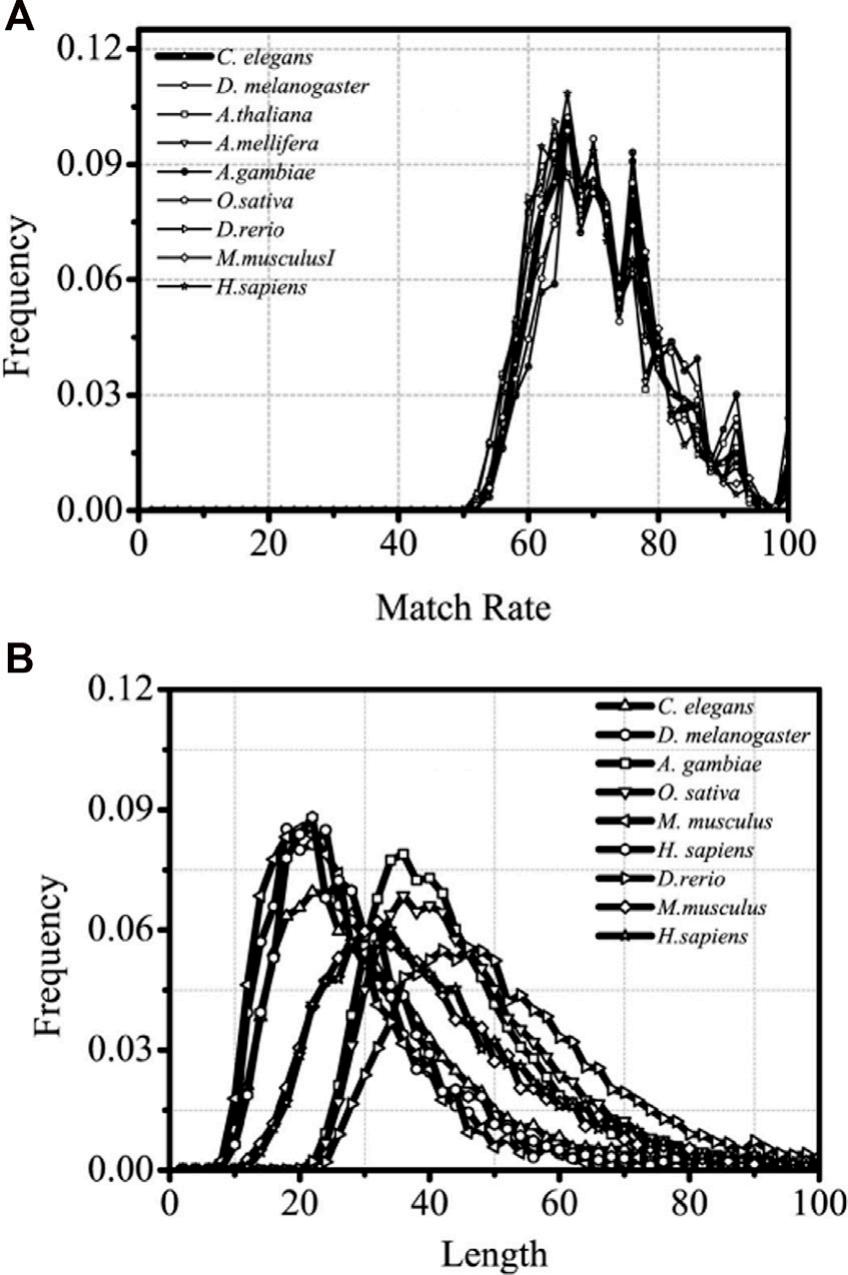
Matched rate and Length distributions distributions of different intron optimal matchedsegments, separately. The X-axis is Length and matched rate (%)of intron optimal matched segment,separately and the Y-axis represents the Frequency values.

It is of great significance when the interaction between introns and the corresponding coding sequences are studied and the sequence characteristics of the optimal matched fragments are analyzed. The sequence paired rate and distribution length of the optimal matched segments of introns between the introns and corresponding mRNA are explored in this section. The results are shown in Figure 3.

From lower eukaryotes to higher eukaryotes, the high consistency of the matched rate of distribution of the optimal matched segments is seen, and the matched rate fluctuates between 60% and 80%. Several clear and conservative peaks are observed, with a clear maximal peak at about 68% and a distinctly sub-maximal peak at about 75%, followed by several discrete peaks with a gradual decrease in distribution (Figure 3).

It is inferred that introns have a precise “quantum state” with the sequences of the optimal matched mRNA segments for each corresponding intron and that each “quantum state” might represent a specific kind of pattern group in which introns control gene expressions. There is no doubt that this distribution is universal from lower eukaryotes to higher eukaryotes. The optimal matched segments between introns and their associated mRNAs have a very noticeable peak in their sequence length distribution. The most probable value, however, varies between the lower and higher eukaryotes; it is roughly 20 bp for lower eukaryotes and 30 bp for higher eukaryotes. It can be implied that higher and lower eukaryotes may differ significantly in the intricacy of gene expression patterns mediated by introns. When compared with siRNA and miRNA, the optimal matched segment from the introns with the associated mRNA appears to have a beneficial impact on gene expression.

### 3.3 Distributions of optimal matched regions near functional sites

**FIGURE 4:**
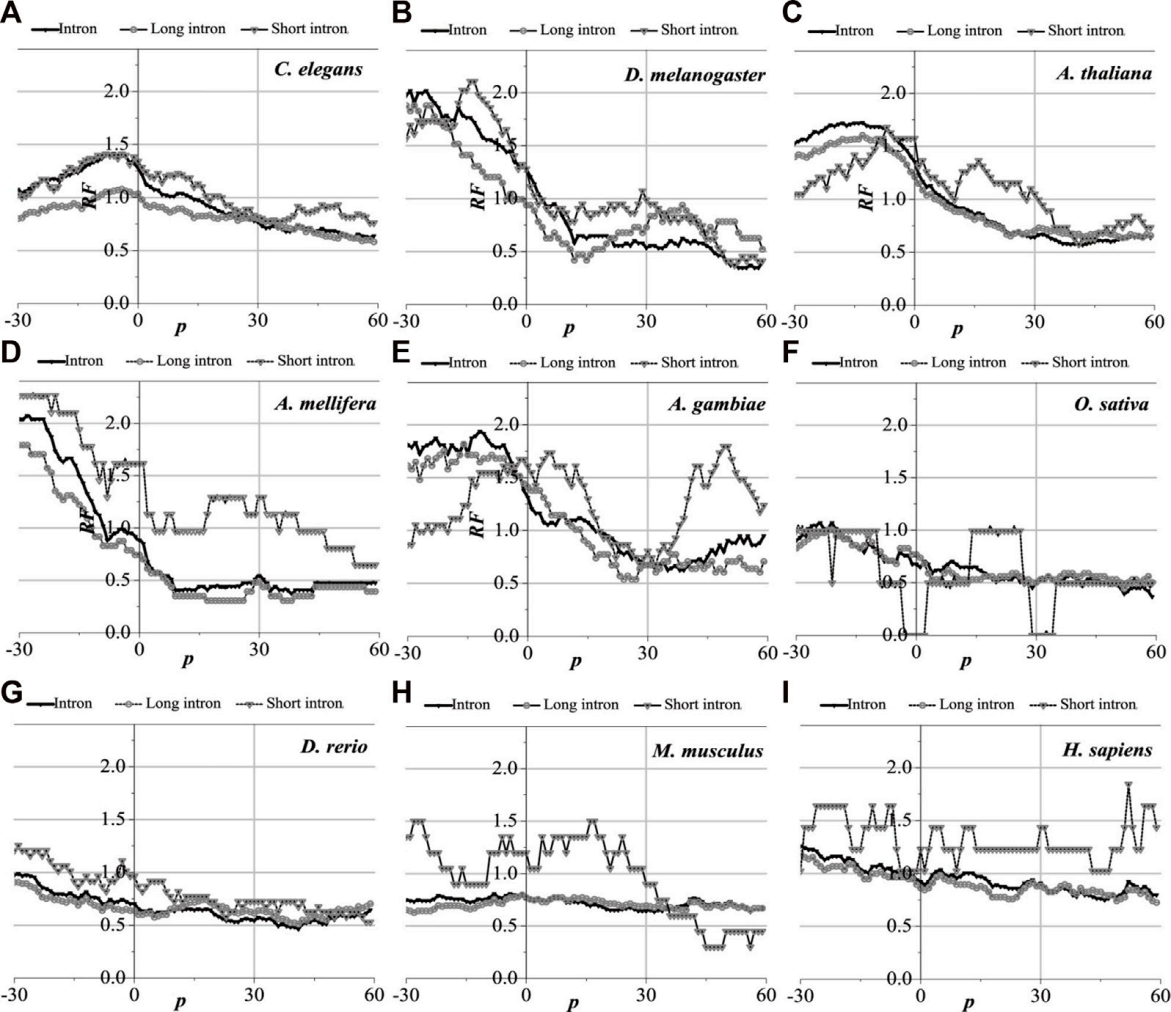
*RF* distributions around translation initiation site. The X-axis is the position of mRNA and the Y-axis represents the *RF* values. *RF* = 1 represents the average value of relative match frequencies theoretically.

**FIGURE 5:**
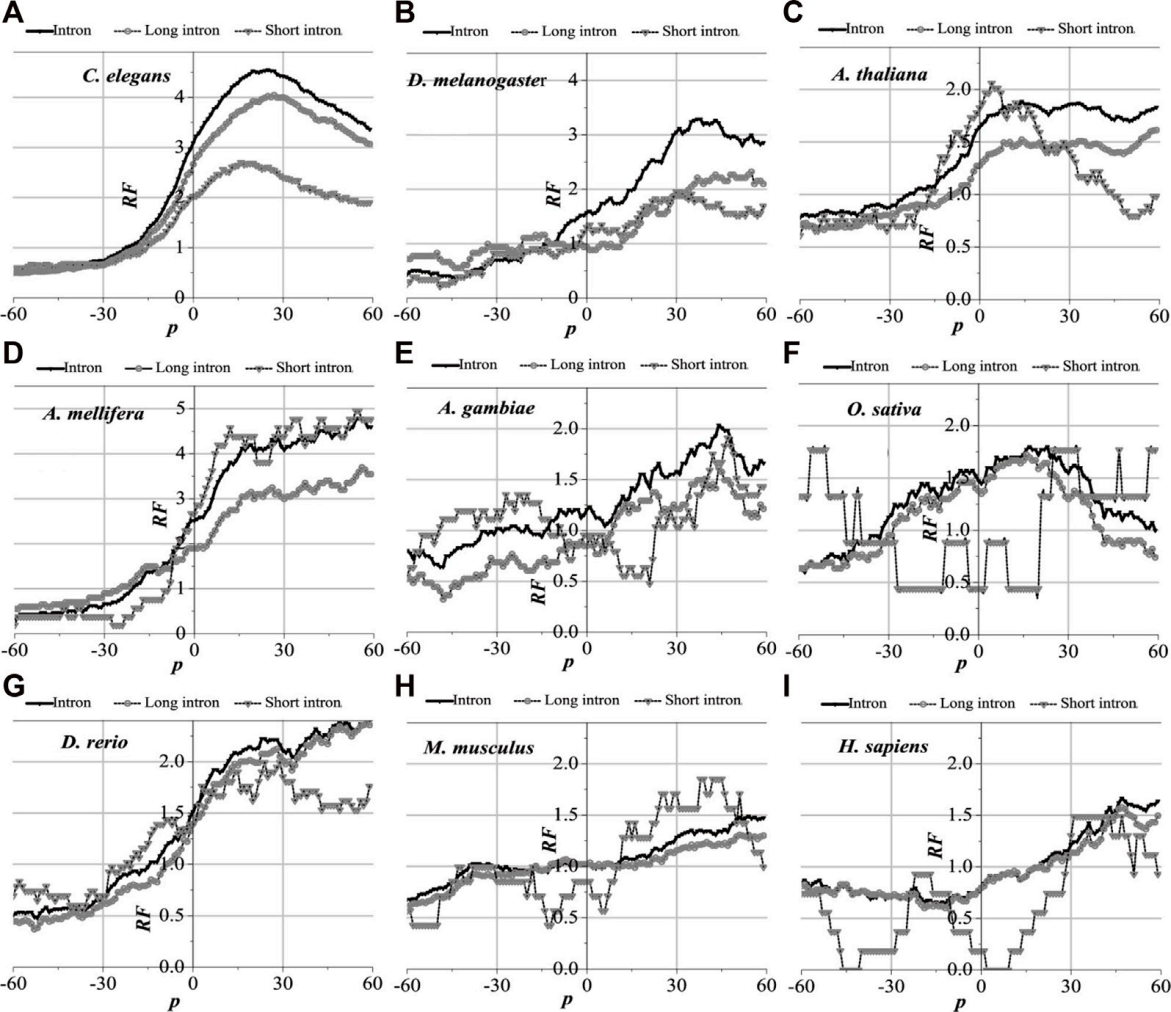
*RF* distributions around translation termination site. The X-axis is the position of mRNA and the Y-axis represents the *RF* values. *RF* = 1 represents the average value of relative match frequencies theoretically.

**FIGURE 6:**
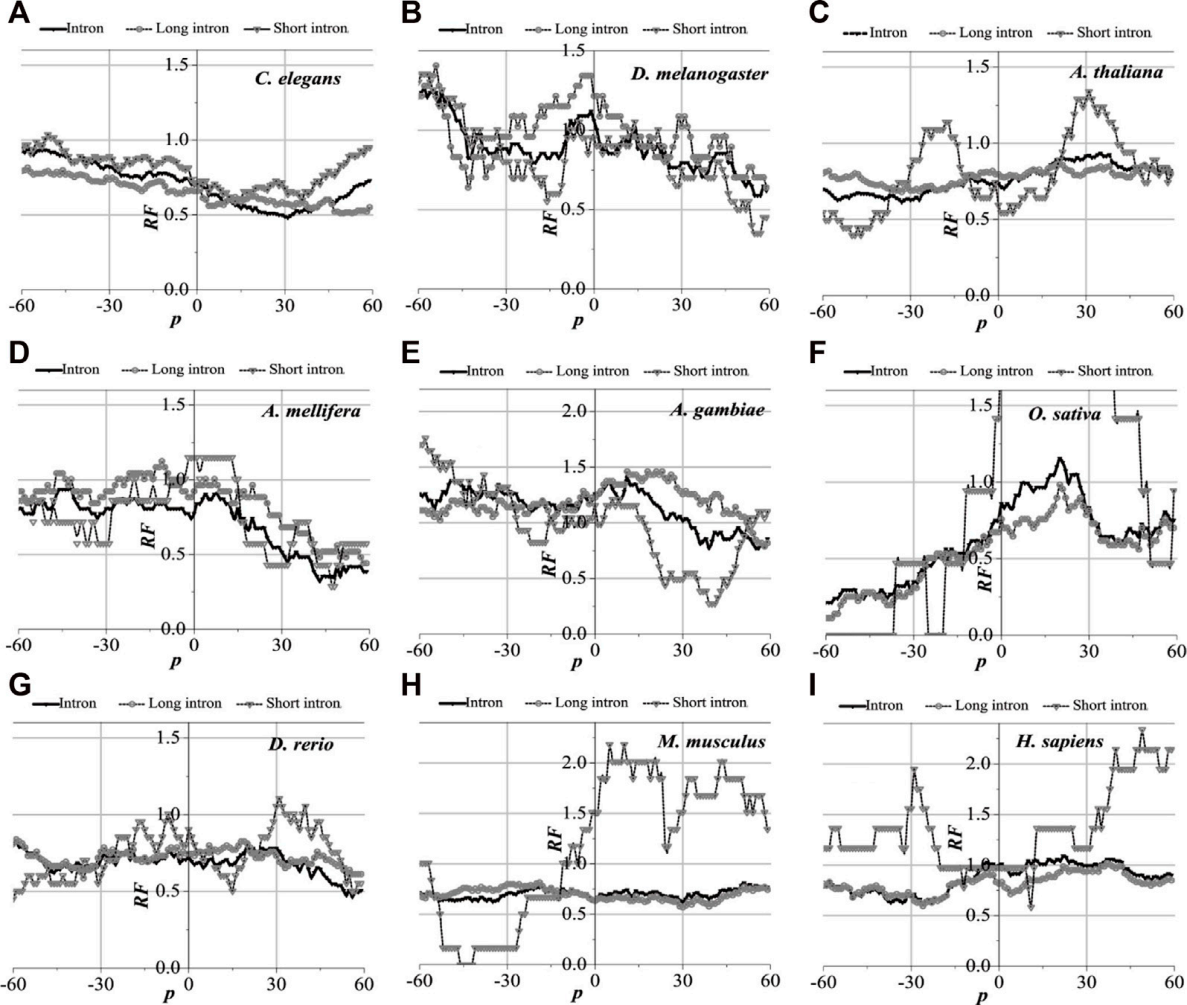
*RF* distributions around the first exon junction site. The X-axis is the position of mRNA and the Y-axis represents the *RF* values. *RF* = 1 represents the average value of relative match frequencies theoretically.

**FIGURE 7:**
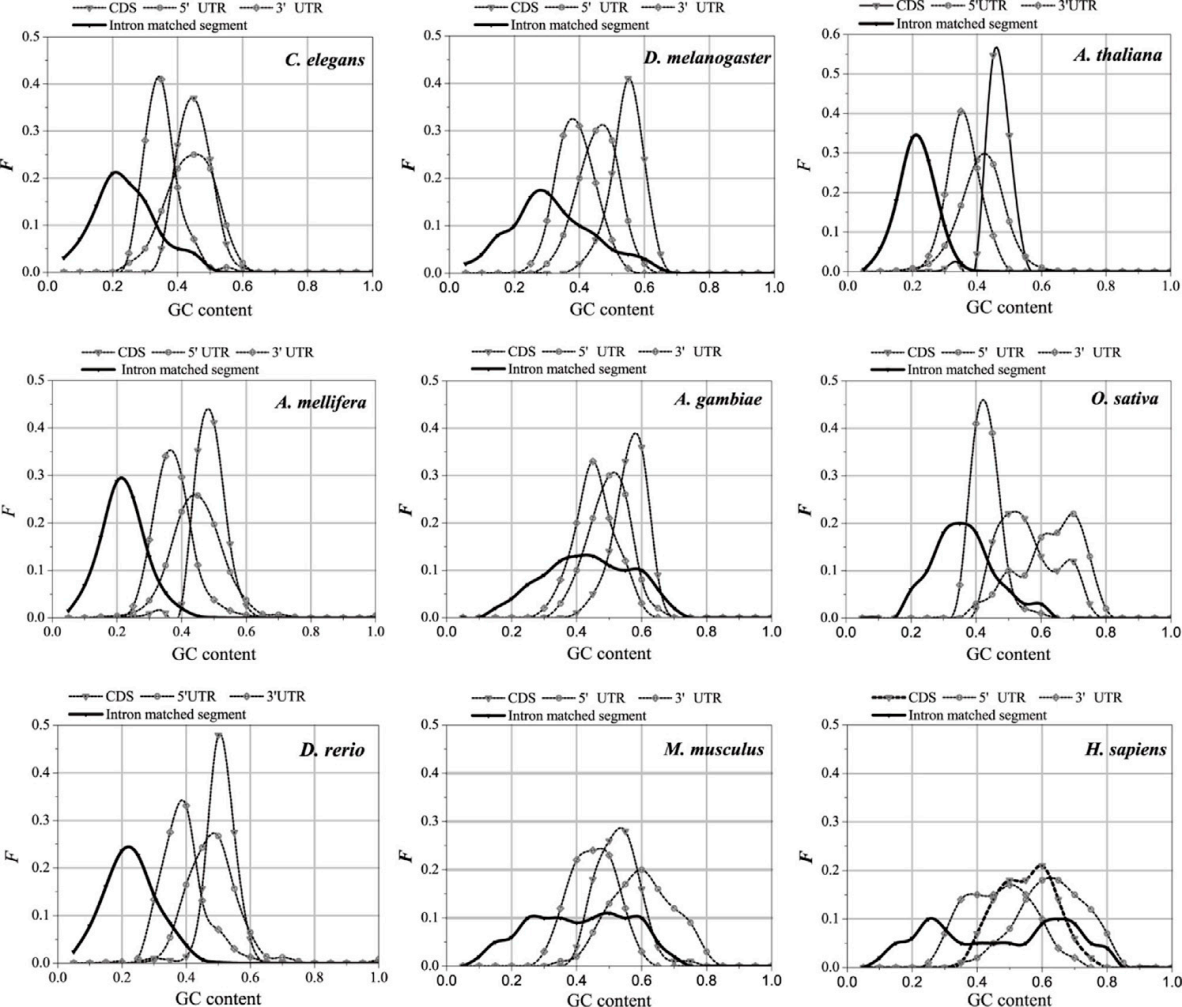
GC content distributions of different sequences. The X-axis is GC content and the Y-axis represents the Frequency values.

Translation initiation sites, translation termination sites, and exon junction sites exert an irreplaceable role in the normal expression of genes. It is particularly crucial to comprehend the distribution of optimal matched frequency near functional locations. The optimal matched intron segments around each functional site containing the UTR gene are separated, functional sites are set as the origin of coordinates, and the distribution law of the optimal matched regions is counted. These functional sites are translation initiation sites, translation termination sites, the junction sites between the first exon and second exon (the first exon junction site), the junction sites between the last exon with the penultimate exon (the last exon junction site), and the junctions in the middle exon (the middle exon junction site). The results are shown in Figures 4–7 (the results of the junction sites Figures 8, 9 are shown in Supplementary Material S5).

#### 3.3.1 Distribution of optimal matched regions of translation initiation regions

Analytically, the distribution of optimal matched frequency of translation initiation sites of the nine model organisms is revealed to be of high consistency, that is, these model species show good universality for the distributions. The matched frequencies bounded by the translation initiation sites on the sequences near the translation initiation site are significantly altered, it is specifically manifested in the relative matched frequencies of the UTR on the left side of the translation initiation site, and the corresponding intron is generally higher. There is an excellent agreement between their distribution of optimal matched regions and the 5′-end of the mRNA. When comparing short introns, the distribution of their optimal matched region is consistent with that of long introns. Large differences in the distribution of optimal matched regions are observed in the short introns, but some distributions are very peculiar in that they may be significant and deserve to be studied in depth.

The distribution of the peak regions and peak value are slightly different despite having very similar distributions of optimal matched regions on the flanks of the translation initiation sites of the nine model organisms (see Figure 4). For *C. elegans*, the optimal matched regions between the flanks of the translation initiation site of mRNA and its corresponding introns are mainly located in the range of about −30 to 10 bp, and the peak value is about 1.4. The optimal matched regions of *D. melanogaster* are mainly located in the range of about −30 to 5 bp, and the peak value is about 2.0. The optimal matched regions of *A. thaliana* are mainly located in the range of about −30 to 10 bp, and the peak value is about 1.5. The optimal matched regions of *A. mellifera* are mainly located at about −10 bp, and the peak value is about 2.0. The optimal matched regions of *A. gambiae* are mainly located in the left range of about −30 to 5 bp, and the peak value is about 1.9. The optimal matched regions of *O. sativa* are mainly located at about −20 bp, and the peak value is about 1.2. The optimal matched regions of *D. rerio* are mainly located in the left range of about −30 bp, and the peak value is about 1.0. There is no distinctly optimal matched region for *M. musculus.* The optimal matched regions of *H. sapiens* are mainly located in the range of about −45 to −10 bp, and the peak value is about 1.3. These facts demonstrate that introns do interact with the translation initiation sites flanking their corresponding mRNAs.

#### 3.3.2 Distribution of optimal matched regions of translation termination regions

Analytically, the distribution of optimal matched frequency of translation termination sites of the nine model organisms is revealed to be alike, that is, these model species show good universality for this distribution. The matched frequencies bounded by the translation termination sites are significantly altered; it is specifically manifested in the relative matched frequencies of the UTR on the right side of the translation termination sites, where it is generally higher. There is an excellent agreement between their distribution of optimal matched regions and the 3′-end of the mRNA. When comparing short introns, the distribution of their optimal matched region is consistent with that of long introns. Large differences in the distribution of optimal matched regions are observed in the short introns, but some distributions are very peculiar in that they may be significant and deserve to be studied in depth.

The distribution of the peak regions and peak value are slightly different despite being very similar for the distribution of optimal matched regions on the flanks of the translation termination sites of the nine model organisms (Figure 5). The optimal matched regions between the flanks of the translation termination sites of the *C. elegans* mRNA and their corresponding introns are mainly located in the right range of about −20 bp, and the peak value is about 4.5. The optimal matched regions of *D. melanogaster* are mainly located in the right range of about −10 bp, and the peak value is about 3.3. The optimal matched regions of *A. thaliana* are mainly located in the right range of about −20 bp, and the peak value is about 1.8. The optimal matched regions of *A. mellifera* are mainly located in the right range of about −20 bp, and the peak value is about 4.6. The optimal matched regions of *A. gambiae* are mainly located in the right range of about −15 bp, and the peak value is about 1.8. The optimal matched regions of *O. sativa* are mainly located in the range of about −30–60 bp, and the peak value is about 1.6. The optimal matched regions of *D. rerio* are mainly located in the right range of about −18 bp, and the peak value is about 2.6. The optimal matched regions of *M. musculus* are mainly located in the right range of about −10 bp, and the peak value is about 1.5. The optimal matched regions of *H. sapiens* are mainly located in the right range of about 16 bp, and the peak value is about 1.8. These facts demonstrate that introns do interact with the translation termination sites flanked by their corresponding mRNAs.

#### 3.3.3 Distribution of optimal matched regions of exon–exon junction regions

The distribution of the optimal matched regions around the first exon junction sites, the last exon junction sites, and the middle junction sites of the nine model organisms is detected to be alike after analysis, that is, these model species show good universality for this distribution. It is specifically manifested in the generally low relative matched frequencies of the junction flanked by the first exon with the corresponding introns (Figure 6), the last exon junction sites (see Supplementary Figure S1), and the middle junction sites (Supplementary Figure S2). There is an excellent fit between their distributions of the optimal matched regions and CDS regions in all the exon–exon junction regions. When comparing short introns, the distributions of their optimal matched regions are consistent with those of long introns. For short introns, large differences in their distributions of the optimal matched regions are observed, but some distributions are very peculiar in that they may be significant and deserve to be studied in depth.

### 3.4 Sequence feature of optimal matched segment of introns

The UTR regions of the mRNA preferentially interact with the introns, while the CDS regions of the mRNA are poorly matched to the introns. This is probably why the sequence features of the optimal matched segments are similar to the UTR features. GC content and second-order information redundancy *D*_2_ of the optimal matched segment, CDS, 3′-UTR, and 5′-UTR are analyzed, and the correlation between these sequences is discussed. The results are shown in Figure 7 and Table 2.

**TABLE 2:** *D*_*2*_ for different sequences of nine eukaryotes.

	*D*_*2*_			
	CDS	5′-UTR	3′-UTR	Intron matched segment
*Caenorhabditis elegans*	0.029	0.032	0.32	0.066
*Drosophila melanogaster*	0.015	0.023	0.009	0.010
*Arabidopsis thaliana*	0.018	0.026	0.012	0.010
*Apis mellifera*	0.019	0.021	0.009	0.012
*Anopheles gambiae*	0.014	0.025	0.010	0.011
*Oryza sativa*	0.016	0.015	0.015	0.011
*Danio rerio*	0.021	0.028	0.014	0.013
*Mus musculus*	0.042	0.031	0.040	0.046
*Homo sapiens*	0.041	0.028	0.043	0.063

#### 3.4.1 GC content of optimal matched segment of intron

The distributions in Figure 6 are compared, and the GC content distributions of the optimal matched segments, CDS, 3′-UTR, and 5′-UTR in the nine model organisms are analyzed.

There are significant differences in the distribution center of the GC content in different sequences; however, the GC content of the optimal matched segments shows a special distribution pattern. In addition to having the lowest distribution center when compared to the other three types, the GC content distribution also has a very broad distribution range that almost completely encloses the distribution of the other sequences. It is shown that interactions between introns and mRNAare primarily based on weak bond binding, i.e., AT matching, but that high GC matching canalso occur. The average GC content of the optimal matched segments of introns in the nine model organisms is the closest to that of the 3′-UTR. The average GC content of the optimal matched segments, 3′-UTR, 5′-UTR, and CDS of *C. elegans*, *D. melanogaster*, *A. thaliana*, *A. gambiae*, *A. mellifera*, *D. rerio*, and *H. sapiens* increased gradually. The average GC content of the optimal matched segments, 3′-UTR, CDS, and 5′-UTR in *O. sativa* and *M. musculus* increased gradually. It is an interesting case that there are two clear peaks in the average GC content distributions of the optimal matched segments for *A. gambiae*, *M. musculus*, and *H. sapiens*, and these results show that there are abundant functional units in the introns. Based on the GC content analysis results of the above sequences, similar GC content values may lead to the mRNA UTR preference for intron interaction, except for *O. sativa* and *M. musculus*.

#### 3.4.2 Second-order information redundancy of optimal matched segments of introns

The intron of each gene is connected with the optimal matched segments of the mRNA, CDS, 3′-UTR (which includes 50 bp downstream of the translation initiation site), and 5′-UTR (which includes 50 bp upstream of the translation termination site) in a new line in sequence and are denoted as the optimal matched segments of the intron, CDS, 3′-UTR, and 5′-UTR. The results of the second-order information redundancy *D*_*2*_ are shown in Table 2.

By comparing the D_2_ values in Table 2, it is seen that the 3′-UTR D_2_ values are the closest to those of the optimal matched intron segments in the nine model species. This agrees with the GC content of the optimal matched intron segments. This is one of the reasons why there are optimal matched frequent peaks in the regions of UTR, which is obvious, especially in the 3′-UTR.

## 4 Conclusion

At the genome-wide level, the optimal matched regions of the introns and their corresponding mRNAs for protein-coding genes in nine model organisms (such as *H. sapiens*) were analyzed. It was observed that the distribution of optimal matched frequencies in the mRNA sequence showed high consistency or universality among the nine model organisms. A peak distribution appeared in the untranslated regions (UTRs) of the mRNA, especially in the 3′-UTR, and the matched frequency in the coding sequence (CDS) was relatively low. It was discovered that introns, particularly the 3′-UTR, have a high preference for interacting with the UTR region of the mRNA. The function of the introns after splicing could be related to NMD. The matched frequencies bounded by functional sites in the sequences near the translation initiation site and translation termination site were different significantly, and the matched frequency of the junction region of the exon was relatively low. The distribution centers of the GC content in different sequences were different, but the GC content in the optimal matched segments showed a special distribution pattern. In addition to having a lower distribution center than the other three types, the GC content also had a very broad distribution range that almost completely enclosed the distribution of the other sequences. The results showed that the interactions between the introns and mRNAs were mainly dominated by weak bond binding, that is, not only AT matching but also in juggling high GC matching. In all nine species, a high degree of concordance of the distribution of the matched rate of the optimal matched segments was observed, primarily falling between 60% and 80%. In lower eukaryotes and higher eukaryotes, the most probable value of the optimal matched segment length distribution is around 20 bp and around 30 bp, respectively. These conclusions are in line with the results obtained in the ribonucleoprotein genes. Some peaks of the distribution of matched frequency are conserved for all organisms, and the results reveal the inherent mechanisms of the optimal matched segment composition.

## Data Availability

The original contributions presented in the study are included in the article/Supplementary Material; further inquiries can be directed to the corresponding authors.

## References

[B1] Bianchi M. Crinelli R. Giacomini E. Carloni E. Magnani M. (2009). A potent enhancer element in the 5'-UTR intron is crucial for transcriptional regulation of the human ubiquitin C gene. Gene 448 (1), 88–101. 10.1016/j.gene.2009.08.013 19733223

[B35] Bo S. Li H. Zhang Q. Lu Z. Bao T. (2019). Potential relations between post-spliced introns and mature mRNAs in the Caenorhabditis elegans genome. J. Theor. Biol. 467, 7-14. 10.1016/j.jtbi.2019.01.031 30710554

[B2] Braddock M. Muckenthaler M. White M. R. Thorburn A. M. Sommerville J. Kingsman A. J. (1994). Intron-less RNA injected into the nucleus of Xenopus oocytes accesses a regulated translation control pathway. Nucleic Acids Res. 22 (24), 5255–5264. 10.1093/nar/22.24.5255 7816614 PMC332069

[B3] Brinster R. L. Allen J. M. Behringer R. R. Gelinas R. E. Palmiter R. D. (1988). Introns increase transcriptional efficiency in transgenic mice. Proc. Natl. Acad. Sci. 85 (3), 836–840. 10.1073/pnas.85.3.836 3422466 PMC279650

[B4] Buchman A. R. Berg P. (1988). Comparison of intron-dependent and intron-independent gene expression. Mol. Cell. Biol. 8, 4395–4405. 10.1128/mcb.8.10.4395-4405.1988 3185553 PMC365513

[B5] Buckley P. T. Lee M. T. Sul J-Y. Miyashiro K. Y. Bell T. J. Fisher S. A. (2011). Cytoplasmic intron sequence-retaining transcripts can be dendritically targeted via ID element retrotransposons. Neuron 69 (5), 877–884. 10.1016/j.neuron.2011.02.028 21382548 PMC3065018

[B6] Callis J. Fromm M. Walbot V. (1987). Introns increase gene expression in cultured maize cells. Genes and Dev. 1 (10), 1183–1200. 10.1101/gad.1.10.1183 2828168

[B7] Charital Y. M. Haasteren G. Massiha A. Schlegel W. Fujita T. (2009). A functional NF-kappaB enhancer element in the first intron contributes to the control of c-fos transcription. Gene 430 (1-2), 116–122. 10.1016/j.gene.2008.10.014 19026727

[B8] Christie M. Croft L. J. Carroll B. J. (2011). Intron splicing suppresses RNA silencing in Arabidopsis. Plant J. 68 (1), 159–167. 10.1111/j.1365-313X.2011.04676.x 21689169

[B9] Daehyun B. Phil G. (2005). Sequence conservation, relative isoform frequencies, and nonsense-mediated decay in evolutionarily conserved alternative splicing[J]. Proc. Natl. Acad. Sci. U. S. A. 102 (36), 12813. 10.1073/pnas.0506139102 16123126 PMC1192826

[B10] Duncker B. Davies P. Walker V. (1997). Introns boost transgene expression in *Drosophila melanogaster* . Mol. General Genet. MGG 254 (3), 291–296. 10.1007/s004380050418 9150263

[B11] Duret L. (2001). Why do genes have introns Recombination might add a new piece to the puzzle [J]. TRENDS Genet. 17 (4), 172–175. 10.1016/s0168-9525(01)02236-3 11275306

[B12] Gozashti L. Roy S. W. Thornlow B. Kramer A. Ares M. Corbett-Detig R. (2022). Transposable elements drive intron gain in diverse eukaryotes[J]. Proc. Natl. Acad. Sci. U. S. A. 119 (48), e2209766119. 10.1073/pnas.2209766119 36417430 PMC9860276

[B13] Guigó R. Ullrich S. (2020). Dynamic changes in intron retention are tightly associated with regulation of splicing factors and proliferative activity during B-cell development. Nucleic Acids Res. 48 (3), 1327–1340. 10.1093/nar/gkz1180 31879760 PMC7026658

[B14] Halligan D. L. Keightley P. D. (2006). Ubiquitous selective constraints in the Drosophila genome revealed by a genome-wide interspecies comparison. Genome Res. 16 (7), 875–884. 10.1101/gr.5022906 16751341 PMC1484454

[B15] Hansen T. B. Wiklund E. D. Bramsen J. B. Villadsen S. B. Statham A. L. Clark S. J. (2011). miRNA-dependent gene silencing involving Ago2-mediated cleavage of a circular antisense RNA[J]. EMBO J. 30 (21), 4414. 10.1038/emboj.2011.359 21964070 PMC3230379

[B16] Hir H. L. Nott A. Moore M. J. (2003). How introns influence and enhance eukaryotic gene expression. Trends Biochem. Sci. 28 (4), 215–220. 10.1016/S0968-0004(03)00052-5 12713906

[B17] Jeck W. R. Sorrentino J. A. Wang K. Slevin M. K. Burd C. E. Liu J. (2013). Circular RNAs are abundant, conserved, and associated with ALU repeats[J]. RNA, 19. 141. 10.1261/rna.035667.112 23249747 PMC3543092

[B18] Julia S. Charles G. Lincoln W. P. Lacayo N. Brown P. O. (2012). Circular RNAs are the predominant transcript isoform from hundreds of human genes in diverse cell types[J]. PloS one 7 (2), e0030733. 10.1371/journal.pone.0030733 PMC3270023 22319583

[B19] Ko C. H. Brendel V. Taylor R. D. Walbot V. (1998). U-richness is a defining feature of plant introns and may function as an intron recognition signal in maize. Plant Mol. Biol. 36 (4), 573–583. 10.1023/a:1005932620374 9484452

[B32] Li W. (1990). Mutual information functions versus correlation functions. J. Stat. Phys. 60 (5-6), 823–837. 10.1007/BF01025996

[B33] Luo (2004). Theoretic-physical approach to molecular biology (1 ref). Shanghai Scientific and Technical Publishers.

[B34] Luo L. Hong L. (1991). The statistical correlation of nucleotides in protein-coding DNA sequences. Bull. Math. Biol. 53 (3), 345–353. 1863813 10.1007/BF02460722

[B20] Luo M. J. Reed R. (1999). Splicing is required for rapid and efficient mRNA export in metazoans. Proc. Natl. Acad. Sci. 96 (26), 14937–14942. 10.1073/pnas.96.26.14937 10611316 PMC24751

[B21] Matsumoto K. Wassarman K. M. Wolffe A. P. (1998). Nuclear history of a pre-mRNA determines the translational activity of cytoplasmic mRNA. EMBO J. 17 (7), 2107–2121. 10.1093/emboj/17.7.2107 9524132 PMC1170555

[B22] Mattick J. S. Gagen M. J. (2001). The evolution of controlled multitasked gene networks: The role of introns and other noncoding RNAs in the development of complex organisms. Mol. Biol. Evol. 18 (9), 1611–1630. 10.1093/oxfordjournals.molbev.a003951 11504843

[B23] McKenzie R. W. Brennan M. D. (1996). The two small introns of the Drosophila affinidisjuncta Adh gene are required for normal transcription. Nucleic Acids Res. 24 (18), 3635–3642. 10.1093/nar/24.18.3635 8836194 PMC146134

[B24] Nordin A. Larsson E. Holmberg M. (2012). The defective splicing caused by the ISCU intron mutation in patients with myopathy with lactic acidosis is repressed by PTBP1 but can be derepressed by IGF2BP1. Hum. Mutat. 33 (3), 467–470. 10.1002/humu.22002 22125086

[B25] Nott A. Meislin S. H. Moore M. J. (2003). A quantitative analysis of intron effects on mammalian gene expression. Rna 9 (5), 607–617. 10.1261/rna.5250403 12702819 PMC1370426

[B26] Orphanides G. Reinberg D. (2002). A unified theory of gene expression. Cell 108 (4), 439–451. 10.1016/s0092-8674(02)00655-4 11909516

[B27] Rafiq M. Suen C. K. Choudhury N. Joannou C. L. White K. N. Evans R. W. (1997). Expression of recombinant human ceruloplasmin-an absolute requirement for splicing signals in the expression cassette. FEBS Lett. 407 (2), 132–136. 10.1016/s0014-5793(97)00325-6 9166886

[B28] Roy S. W. (2003). Large-scale comparison of intron positions in mammalian genes shows intron loss but no gain [J]. Proc. Natl. Acad. Sci. 100 (12), 7158–7162. 10.1073/pnas.1232297100 12777620 PMC165846

[B29] Ryu W. S. Mertz J. E. (1989). Simian virus 40 late transcripts lacking excisable intervening sequences are defective in both stability in the nucleus and transport to the cytoplasm. J. virology 63 (10), 4386–4394. 10.1128/JVI.63.10.4386-4394.1989 2550672 PMC251056

[B30] Sebastian M. Marvin J. Antigoni E. Torti F. Krueger J. Rybak A. (2013). Circular RNAs are a large class of animal RNAs with regulatory potency[J]. Nature 495 (7441), 333. 10.1038/nature11928 23446348

[B31] Stover D. A. Verrelli B. C. (2010). Comparative vertebrate evolutionary analyses of type I collagen: Potential of COL1a1 gene structure and intron variation for common bone-related diseases. Mol. Biol. Evol. 28 (1), 533–542. 10.1093/molbev/msq221 20724381

[B40] Zhao X. Li H. Bao T. (2013). Analysis on the interaction between post-spliced introns and corresponding protein coding sequences in ribosomal protein genes. J. Theor. Biol. 328, 33–42. 10.1016/j.jtbi.2013.03.002 23499990

[B41] Zhang Q. Li H. Zhao X. Zheng Y. Meng H. (2016). Analysis on the preference for sequence matching between mRNA sequences and the corresponding introns in ribosomal protein genes. J. Theor. Biol. 392, 113–121. 10.1016/j.jtbi.2015.12.003 26707402

