# Supplementary figures and images for: Ubiquitous conservative interaction patterns between post-spliced introns and their mRNAs revealed by genome-wide interspecies comparison
Source: Front Genet. 2023 Apr 12;14:1151703. doi: 10.3389/fgene.2023.1151703 (PMC10132729; doi:10.3389/fgene.2023.1151703)

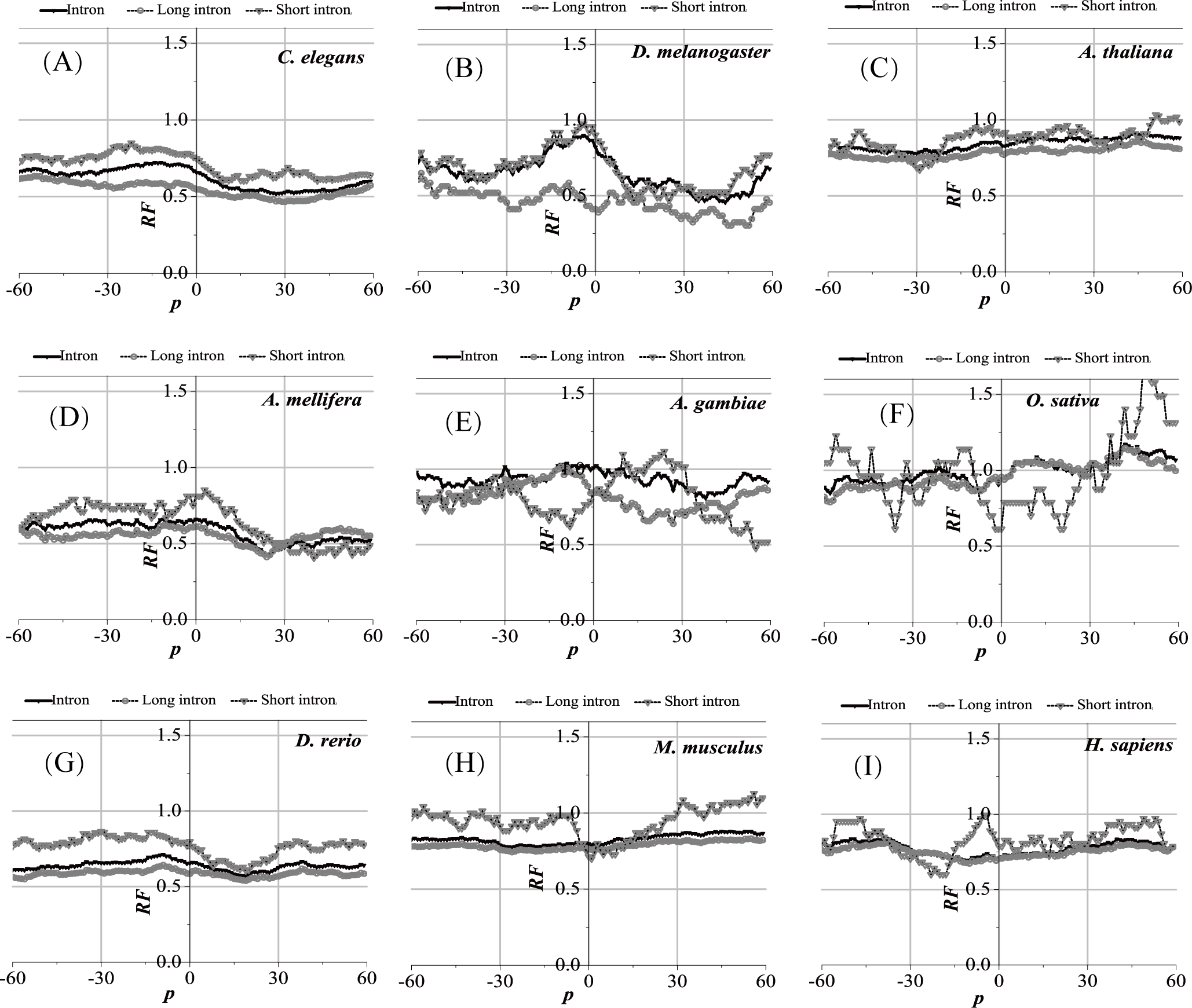

Supplement: Supplementary file 1 [file Presentation1.zip › 1151703_SupMaterial/figure 8.TIF]

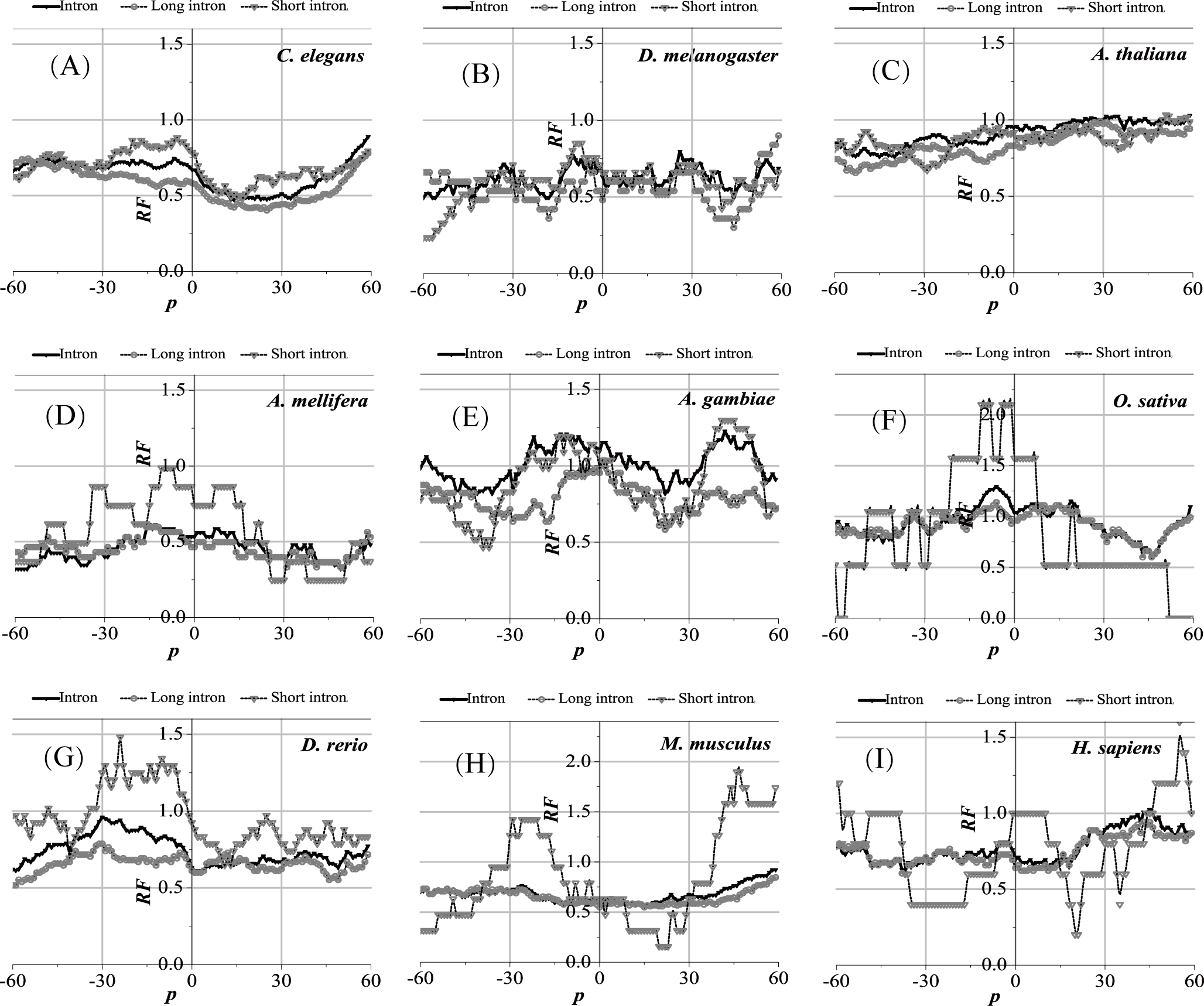

Supplement: Supplementary file 1 [file Presentation1.zip › 1151703_SupMaterial/figure 9.TIF]
