# Supplementary material for: Ubiquitous conservative interaction patterns between post-spliced introns and their mRNAs revealed by genome-wide interspecies comparison
Source: Front Genet. 2023 Apr 12;14:1151703. doi: 10.3389/fgene.2023.1151703 (PMC10132729; doi:10.3389/fgene.2023.1151703)
Supplement: Supplementary file 2 [file Presentation2.pdf]

Supplementary material figure

Figure 8:

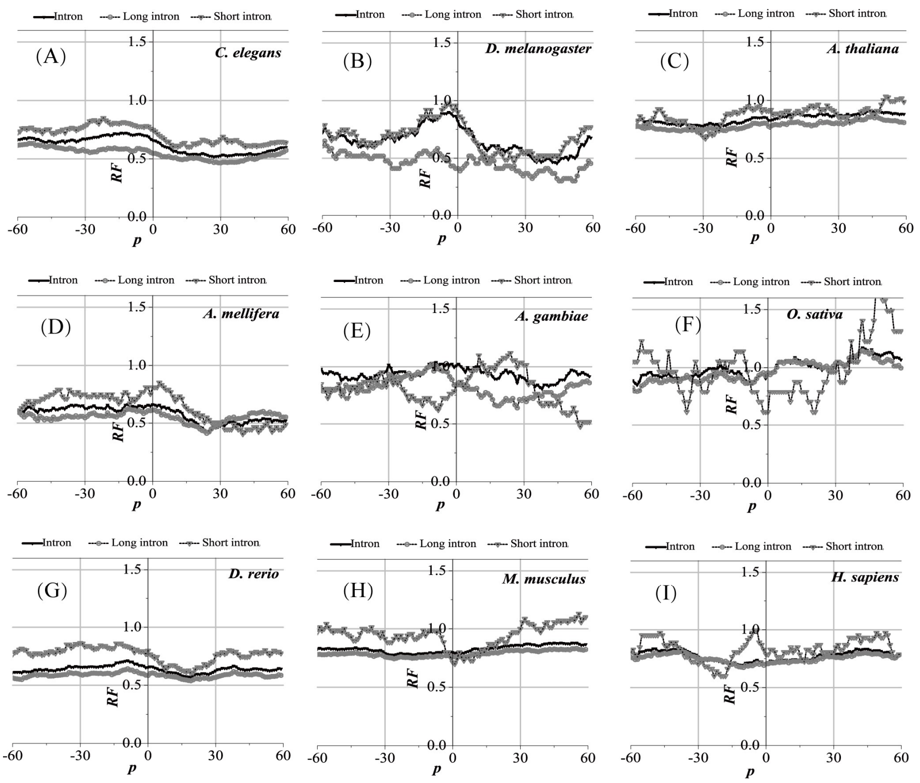

Figure 8 *RF* distributions around the last exon junction site. The X-axis is the position of mRNA and the Y-axis represents the *RF* values. *RF*=1 represents the average value of relative match frequencies theoretically.

Figure 9:

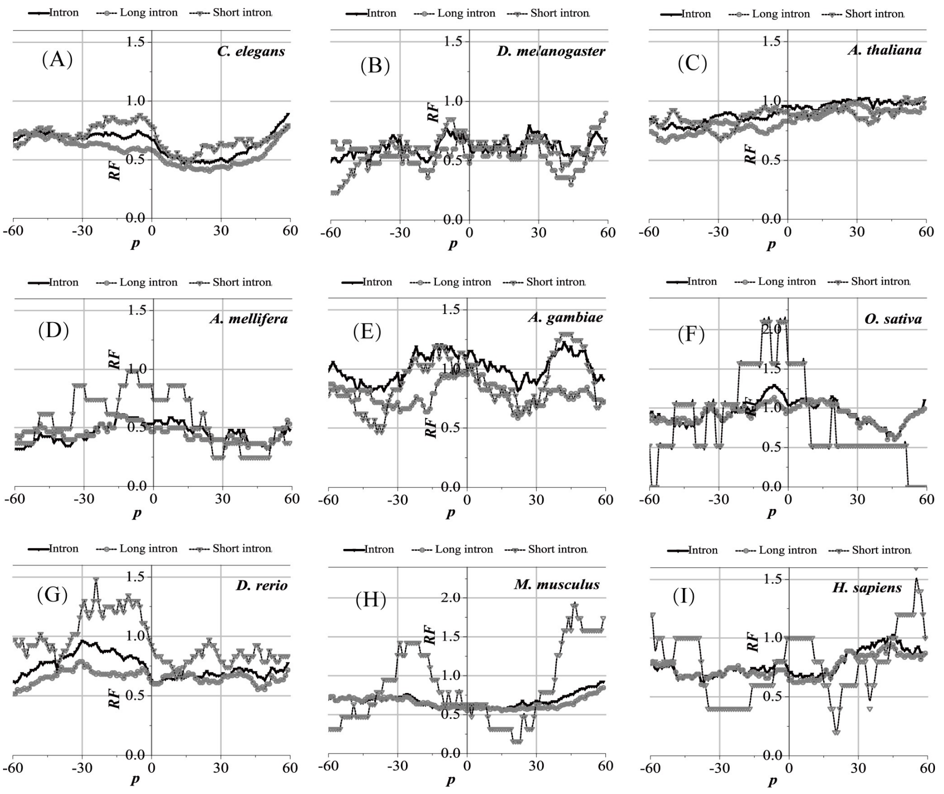

Figure 9 *RF* distributions around the middle exon junction site. The X-axis is the position of mRNA and the Y-axis represents the *RF* values. *RF*=1 represents the average value of relative match frequencies theoretically.
