# Supplementary material for: Ubiquitous conservative interaction patterns between post-spliced introns and their mRNAs revealed by genome-wide interspecies comparison
Source: Front Genet. 2023 Apr 12;14:1151703. doi: 10.3389/fgene.2023.1151703 (PMC10132729; doi:10.3389/fgene.2023.1151703)
Supplement: Supplementary file 3 [file Presentation3.pdf]

Graph and Corresponding Graph Annotation

Figure 1:

```
(a)                 bp                                  bp
     cIntron10  1123    AGGAUCCGGGAAUCAGACUAUAG    1145
                        |.||.||||..||||||||.|.|
     mRNA       798     AUGAUCCGGAGAUCAGACUGUGG    820
(b)
     Intron10   1123    UCCUAGGCCCUUAGUCUGAUAUC    1145
                        |.||.||||..||||||||.|.|
     mRNA       798     AUGAUCCGGAGAUCAGACUGUGG    820
```

Figure 1 Sketch matched map between intron and corresponding CDS. (a) Smith-Waterman local alignment. cIntron10 means complementary segment of intron10. (b) Authentic matched alignment.

Figure 2:

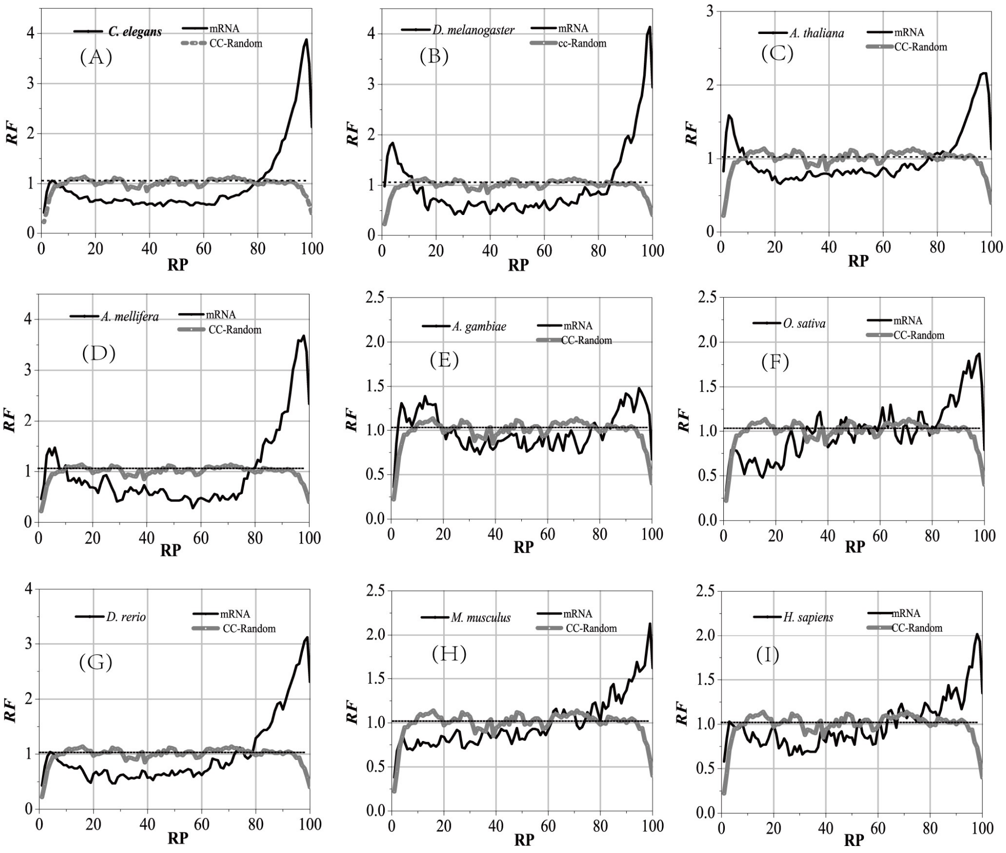

Figure 2 *RF* distributions of mRNA. The X-axis is the relative position of mRNA and the Y-axis represents the *RF* values. CC-Random means the local alignment were done between the component constraint random mRNA and their own component constraint random introns. *RF*=1 represents the average value of relative match frequencies theoretically.

Figure 3:

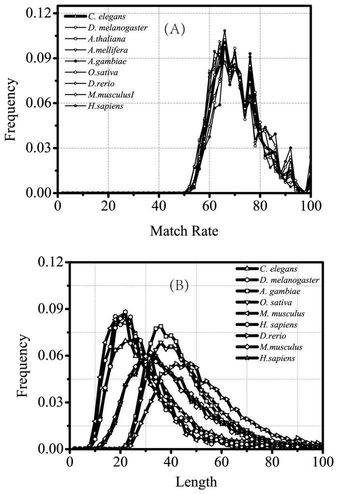

Figure 3 Matched rate and Length distributions distributions of different intron optimal matched segments, separately. The X-axis is Length and matched rate (%)of intron optimal matched segment, separately and the Y-axis represents the Frequency values.

Figure 4:

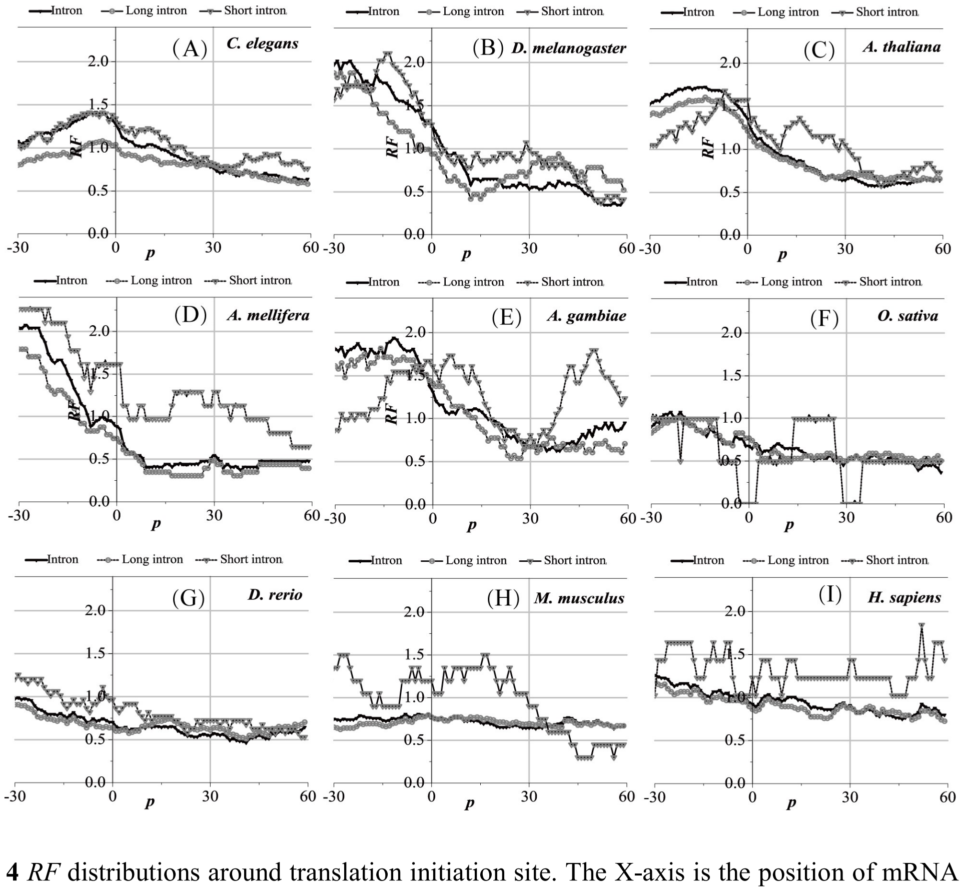

Figure 4 *RF* distributions around translation initiation site. The X-axis is the position of mRNA and the Y-axis represents the *RF* values. *RF*=1 represents the average value of relative match frequencies theoretically.

Figure 5:

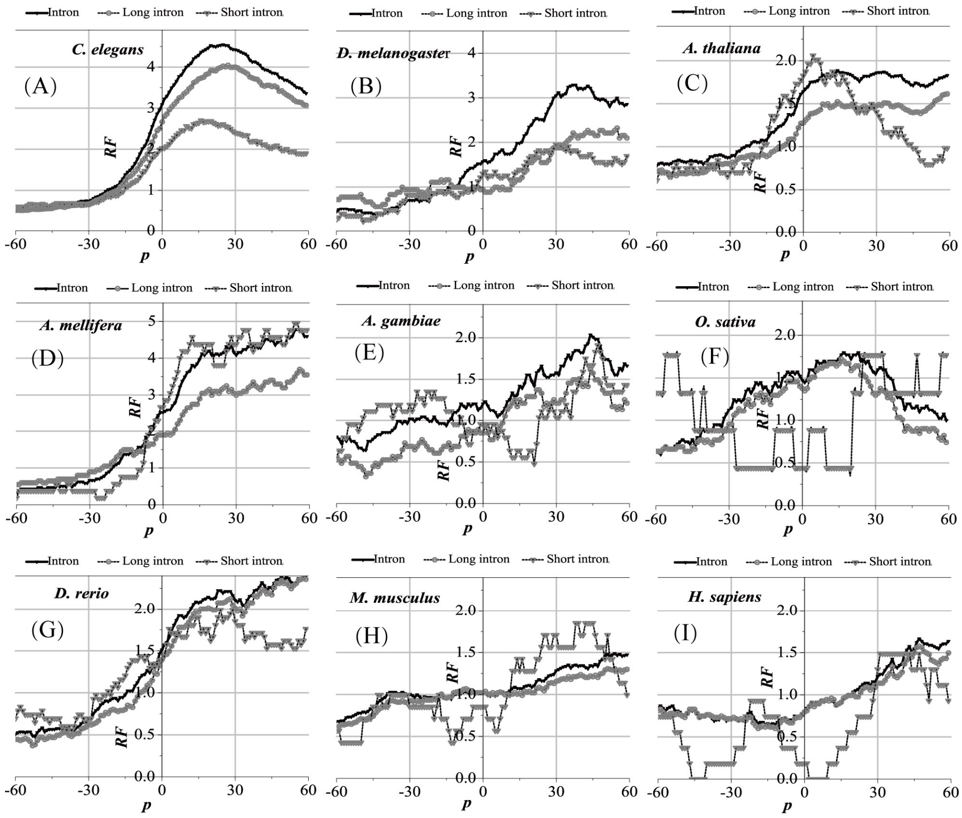

Figure 5 *RF* distributions around translation termination site. The X-axis is the position of mRNA and the Y-axis represents the *RF* values. *RF*=1 represents the average value of relative match frequencies theoretically.

Figure 6:

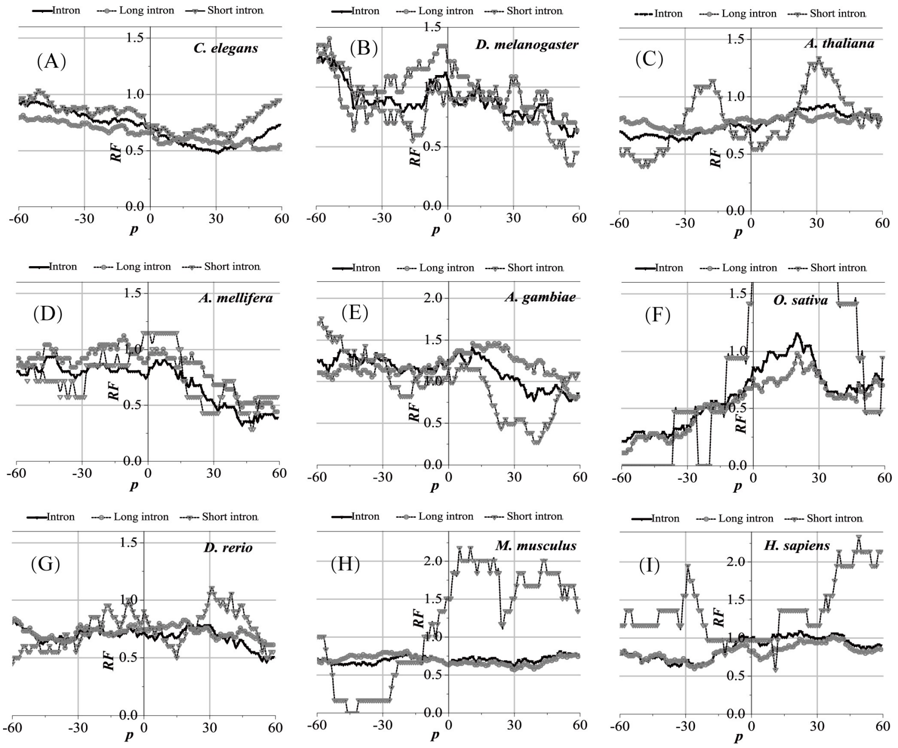

Figure 6 *RF* distributions around the first exon junction site. The X-axis is the position of mRNA and the Y-axis represents the *RF* values. *RF*=1 represents the average value of relative match frequencies theoretically.

Figure 7:

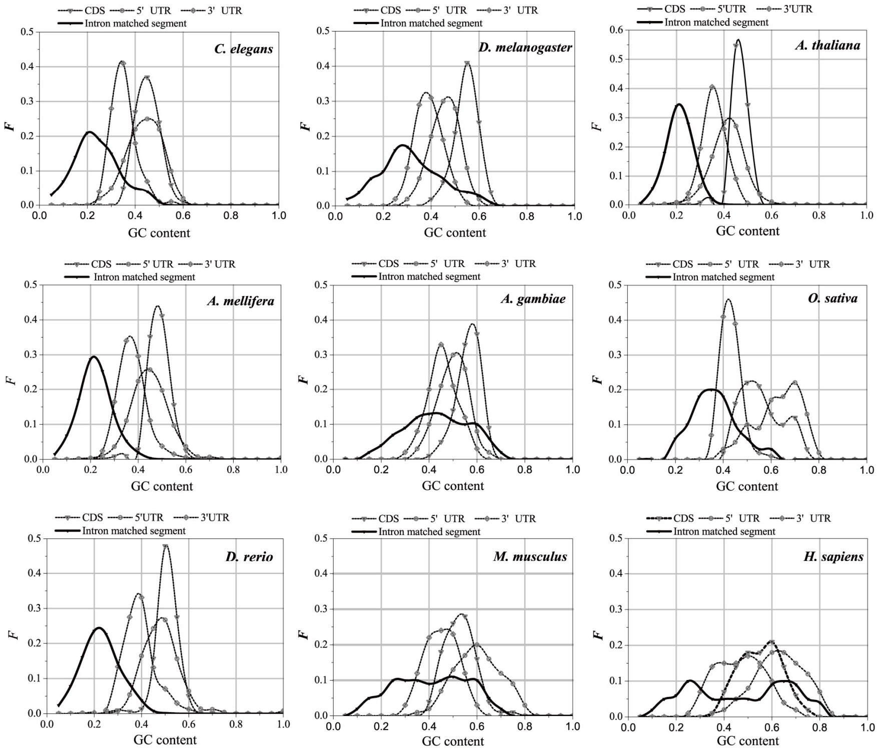

Figure 7 GC content distributions of different sequences. The X-axis is GC content and the Y-axis represents the Frequency values.
